# Role of gut microbiota in the pathogenesis and treatment of diabetes mullites: Advanced research-based review

**DOI:** 10.3389/fmicb.2022.1029890

**Published:** 2022-10-19

**Authors:** Junjun Ye, Zezhen Wu, Yifei Zhao, Shuo Zhang, Weiting Liu, Yu Su

**Affiliations:** ^1^Department of Endocrine and Metabolic Diseases, Longhu Hospital, The First Affiliated Hospital of Shantou University Medical College, Shantou, China; ^2^Shantou University Medical College, Shantou, China; ^3^The First Affiliated Hospital of Shantou University Medical College, Shantou, China; ^4^School of Nursing, Anhui University of Chinese Medicine, Hefei, Anhui, China; ^5^Center of Teaching Evaluation and Faculty Development, Anhui University of Chinese Medicine, Hefei, Anhui, China

**Keywords:** gut microbiota, type 1 diabetes mullites, type 2 diabetes mullites, gut leakiness, gut microbiota metabolites, microbiological therapy

## Abstract

Gut microbiota plays an important role in the proper functioning of human organisms, while its dysbiosis is associated with disease in various body organs. Diabetes mellitus (DM) is a set of heterogeneous metabolic diseases characterized by hyperglycemia caused by direct or indirect insulin deficiency. There is growing evidence that gut microbiota dysbiosis is closely linked to the development of DM. Gut microbiota composition changes in type 1 diabetes mullites (T1DM) and type 2 diabetes mullites (T2DM) patients, which may cause gut leakiness and uncontrolled entry of antigens into the circulation system, triggering an immune response that damages the isle β cells or metabolic disorders. This review summarizes gut microbiota composition in healthy individuals and compares it to diabetes mullites patients. The possible pathogenesis by which gut microbiota dysbiosis causes DM, particularly gut leakiness and changes in gut microbiota metabolites is also discussed. It also presents the process of microbial-based therapies of DM.

## Introduction

**Figure 1:**
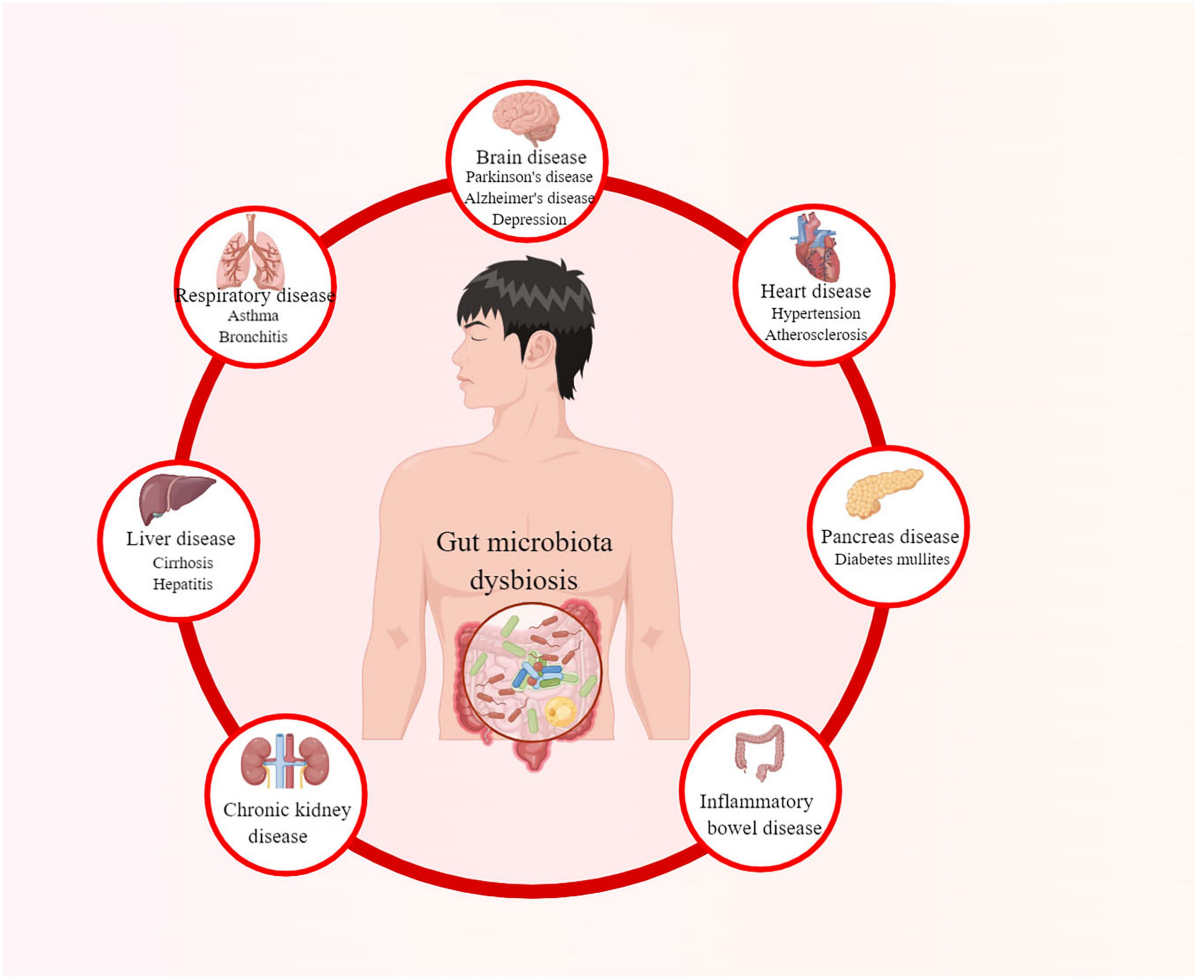
Diseases caused by gut microbiota dysbiosis.

The gut microbiota plays an essential role in the proper functioning of human organisms (Vrancken et al., 2019). It co-evolves and symbioses with humans by combating pathogenic bacteria, assisting nutrient digestion, maintaining the integrity of the intestinal epithelia, and promoting immunological development (Stecher and Hardt, 2011; Dave et al., 2012; Natividad et al., 2012; Shreiner et al., 2015). However, when the balance of the microbiota community is affected, known as gut microbiota dysbiosis, it may lead to various diseases, as summarized in Figure 1 (Hou et al., 2022a). Diabetes mullites (DM) is a set of heterogeneous metabolic disorders characterized by hyperglycemia and glucose intolerance, with high and increasing prevalence and multiple complications (Danaei et al., 2011; Saeedi et al., 2019; Classification and Diagnosis of Diabetes, 2022). The classical opinion is that type 1 diabetes mullites (T1DM) results from autoreactive T-cell-mediated partially or absolute destruction of pancreatic β cells in patients (Atkinson et al., 2014). In contrast, type 2 diabetes mullites (T2DM) is the outcome of a progressive loss of sufficient pancreatic β-cell insulin secretion in the context of insulin resistance (IR) (Kahn et al., 2014). According to International Diabetes Federation, there were more than 463 million DM patients in 2019 over the world and this number is estimated to rise to 578 million by 2030 and even 700 million by 2045 (Saeedi et al., 2019).

In the past decade, studies on the gut microbiome have developed rapidly due to the advancements in sequencing technologies and data analysis. It has been observed that gut microbiota dysbiosis is presented in both T1DM and T2DM patients (Bibbò et al., 2017; Vallianou et al., 2018). Gut microbiota dysbiosis may cause gut leakiness, which leads to external antigens uncontrollably entering the circulatory system (Di Tommaso et al., 2021). These antigens could activate islet autoimmunity and directly damage pancreatic β cells, and gut microbiota metabolites may also cause hormonal effects leading to metabolic disorders (Sun et al., 2015; Zhu and Goodarzi, 2020). Immune and metabolic disorders play an important role in the pathogenesis of DM (Bachem et al., 2019; Hou et al., 2022a). In addition, many diabetic complications are proven to be linked to the gut microbiota, including diabetic retinopathy, diabetes-induced cognitive impairment, diabetic peripheral neuropathy, and diabetic nephropathy (Zhou et al., 2022). Although the role of gut microbiota in the pathogenesis of DM is not yet understood, more and more researchers are pinning new hopes for treating DM in microbiological therapy.

Diabetes mellitus and its complications cause physical and mental injury to patients and a great economic burden to the medical system. However, there is no single treatment that can sustainably and consistently prevent the progression of β-cell failure after the onset of DM. Elucidating the role of gut microbiota in the onset and progression of DM will contribute to a better understanding of DM and the development of new treatments for it. This review summarizes the gut microbiota composition in healthy individuals and compares it with T1DM and T2DM patients. Furthermore, we review the role of gut microbiota dysbiosis in the pathogenesis of DM, particularly gut leakiness, immune disorders, and metabolite disorders. This paper will also present the process of microbial-based therapies of DM in the final.

## Gut microbiota in healthy, T1DM, and T2DM individuals

Gut microbiota has 10 times the number of human cells and 150 times larger gene sets than humans, known as the “human second Genene” (Qin et al., 2010). In healthy individuals, the gut microbiota exhibits high taxonomic diversity, abundant microbial genes, and stable core microbiota (Fan and Pedersen, 2021). Herein, we reviewed the composition of gut microbes in healthy individuals at phylum and genus levels.

At the phyla level, approximately 80–90% of the gut microbiota belongs to *Firmicutes* and *Bacteroidetes*. *Firmicutes* dominate the gut microbiota composition of healthy individuals, while *Bacteroidetes* can favor inflammation by distributing the gut epithelial cells’ barrier function (Tlaskalová-Hogenová et al., 2011). Therefore, *Firmicutes* to *Bacteroidetes* ratio (F/B ratio) is suggested to be a criterion of the health of gut microbiomes (Li and Ma, 2020). In addition, in the human gut, *Actinobacteria*, *Verrucomicrobia* and *Proteobacteria* are the major microbial phyla (Sommer and Bäckhed, 2013; Jandhyala et al., 2015; Landman and Quévrain, 2016). *Actinobacteria*, represented by the *Bifidobacterium* genus, contribute to producing butyrate and inhibiting bacterial translocation (Arboleya et al., 2016). Maintaining diversity in the gut microbiota is essential to keeping healthy, and its dysbiosis is linked to the development of metabolic diseases, including DM (Vallianou et al., 2018). At the genus level, *Bifidobacterium*, *Akkermansia*, *Lachnospira*, *Prevotela* and the butyrate-producing genera, including *Roseburia*, *Faecalibacterium*, *Anaerostipes*, *Subdoligranulum*, and *Eubacterium*, are abundant gut microbiota in healthy individuals (Mokhtari et al., 2021).

Notably, the human gut microbiota composition is not immutable but varies due to individual differences, age, and environmental factors. *Akkermansia muciniphila*, *Veillonella*, *Bacteroides*, *Clostridium botulinum* spp. and *Clostridium coccoides spp*. dominate the diversity of children’s microbiota until a stable gut microbiota is formed (Amabebe et al., 2020). The gut microbiota alters rapidly in the first 2 years of life, matures around age three, and remains relatively stable (Isolauri, 2012; Yatsunenko et al., 2012; Durazzo et al., 2019). At maturity, *Firmicutes*, *Bacteroidetes*, and *Actinobacteria* become the dominant gut microbiota in healthy individuals (Yatsunenko et al., 2012). *Bifidobacterium* tends to decrease in older adults, while *Clostridium* and *Proteobacteria* tend to increase (Guigoz et al., 2008).

### Gut microbiota dysbiosis in T1DM

Previous studies have identified differences in the gut microbiota between healthy individuals and T1DM patients (Bibbò et al., 2017). The stability, connectivity, abundance, and composition of the gut microbiota are probably linked to the development of T1DM (Han et al., 2018). Decreased microbiota diversity is a common gut microbiota shift associated with T1DM development (Leiva-Gea et al., 2018).

At the phyla level, it was well documented that proportions of *Firmicutes* phyla decreased in T1DM patients compared to the healthy individual group (Murri et al., 2013; Kostic et al., 2015; Leiva-Gea et al., 2018), while *Bacteroidetes* abundance increased successively (Alkanani et al., 2015; Pellegrini et al., 2017). Furthermore, previous research indicated that the F/B ratio significantly increased over time in children who eventually progressed to clinical T1DM and T1DM (Murri et al., 2013; Pellegrini et al., 2017; Leiva-Gea et al., 2018). However, research also showed no difference in the F/B ratio between T1DM patients and healthy individuals (Qi et al., 2016). Studies assessing the relationship between *Proteobacteria* abundance and T1DM have reported conflicting results, with some reporting a positive association (Brown et al., 2011; Cinek et al., 2018), some reporting a negative association (Leiva-Gea et al., 2018), and some reporting no difference (Murri et al., 2013).

At the genus level, T1DM patients present with a higher abundance of 12 different genera, including *Bifidobacterium*, *Bacteroides*, *Escherichia*, *Veillonella*, *Clostridium*, *Enterobacter*, *Lactobacillus*, *Ruminococcus*, *Streptococcus*, *Sutterella*, *Lactococcus*, and *Blautia*, among which *Bacteroides* is reported to be the dominant genus in the most research literature (Mokhtari et al., 2021). A more recent two-sample Mendelian randomization analysis revealed a close link between a higher relative abundance of the *Bifidobacterium* and a higher risk of T1DM (Xu et al., 2021).

### Gut microbiota dysbiosis in T2DM

Environmental factors play an essential role in the onset and development of T2DM and recent evidence indicates that gut microbiota dysbiosis is one of them (Gurung et al., 2020; Hou et al., 2022b). Like T1DM, the gut microbiota in T2DM differs from those in healthy individuals (Vallianou et al., 2018; Que et al., 2021). The Integrative Human Microbiome Project found that insulin-resistant (IR) individuals had distinguishable molecular and microbial patterns at baseline from the healthy controls group (The Integrative Human Microbiome Project, 2019).

At the phyla level, *Firmicutes* level was reported to be lower in T2DM patients than in healthy individuals, while *Bacteroidetes* level was increased (Larsen et al., 2010). However, some recent research reported the opposite result; *Firmicutes* increased but *Bacteroidetes* decreased in T2DM patients (Sedighi et al., 2017; Zhao et al., 2019). Uniformly, the F/B ratio was reported to increase in some studies, but decreased in some studies (Sedighi et al., 2017; Zhao et al., 2019). In addition, the recent research also indicated an increased level of *Proteobacteria* in T2DM individuals (Sedighi et al., 2017; Zhao et al., 2019).

At the genus level, a large-scale metagenomic analysis in China compared the structural characteristics of the gut microbiota in healthy control and T2DM patients (Qin et al., 2012). It was recognized that conditioned pathogens (*Escherichia coli*, *Bacteroides caccae*, some *Clostridium* species, and *Eggerthella lenta* mainly) were abundant in T2DM patients. On the contrary, the abundance of butyrate-producing gut microbiota (*Roseburia intestinalis*, *Roseburia inulinivorans*, *Eubacterium rectale*, *Faecalibacterium prausnitzii* and Clostridiales sp. *SS3/4*) was decreased. Another large-scale metagenome analysis in Europe demonstrated an increase in the abundance of *Lactobacillus gasseri*, *Streptococcus mutans*, some *Clostridiales* species, and *Lactobacillus* in T2DM patients, while a reduction in the abundance of butyrate-producing microbiota (*Roseburia*, *Eubacterium eligens*, *Bacteroides intestinalis*) (Karlsson et al., 2013). Notably, the above two studies both reported a decrease in butyrate-producing microbiota in T2DM patients, particularly *Roseburia* (Qin et al., 2012; Karlsson et al., 2013). In addition, recent research confirmed the decrease of the *Clostridium* genus in T2DM patients (Allin et al., 2018). Similarly, a lower level of *Akkermansia mucinphila*, which is responsible for degenerating mucin in the gut, was also considered a risk factor for T2DM (Allin et al., 2018; Hasani et al., 2021).

## Role of the gut microbiota in the development of DM

**Figure 2:**
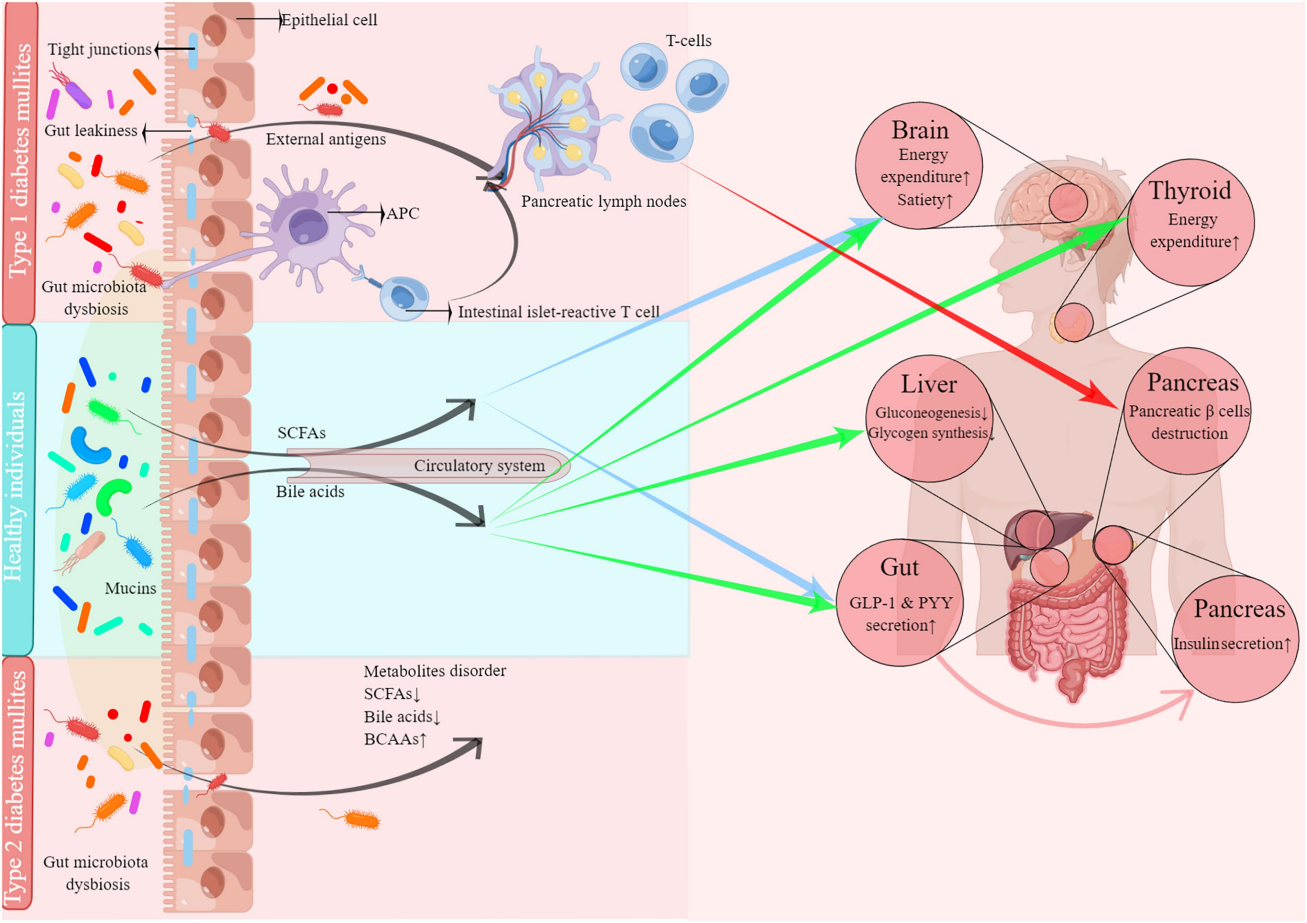
The role of gut microbiota dysbiosis in the development of diabetes mullites.

From the above discussion, we can conclude that DM patients’ gut microbiota is characterized by an increased level of opportunistic pathogens and decreased level of probiotics. Furthermore, *Firmicutes* are negatively associated with T1DM, while *Bacteroidetes* is a positive factor at the phyla level. However, conflicting results have been reported on the difference between healthy individuals and T2DM patients. At the genus level, *Bacteroides* showed a promotive association with both T1DM and T2DM. In addition, both T1DM and T2DM patients presented a lower level of butyrate-producing microbiota. Figure 2 depicts the role of gut microbiota dysbiosis in the development of DM.

### Gut leakiness: A possible origin of DM

An epithelial layer-based physical barrier and a mucosal immune cell-based functional barrier make up the majority of the intestinal barrier and separate the host from the external environment (Mu et al., 2017). Tight junctions (TJ) and mucins are key to maintaining the integrity of the intestinal barrier (Durazzo et al., 2019). Butyrate-producing gut microbiota, such as *Firmicutes*, has been shown to promote TJ assembly, mucin synthesis, and anti-inflammatory properties (Hague et al., 1996; Peng et al., 2007, 2009). On the contrary, *Bacteroides* can inhibit the production of TJ protein and thus promote gut leakiness (Brown et al., 2011; Tlaskalová-Hogenová et al., 2011). Besides, *Bifidobacterium*, *Bacteroides*, and *Ruminococcus* can degrade mucin and thus induce gut leakiness (Hooper et al., 2002). Some bacterial metabolites, such as butyrate, have also been identified to play an important role in maintaining intestinal epithelial integrity (Wang et al., 2012; Kayama et al., 2020). Correspondingly, a low abundance of the butyrate-producing gut microbiota and increased abundance of the *Bacteroides* are presented in DM patients. Of note, hyperglycemia can induce gut leakiness by changing the TJ and adherence junctions (Thaiss et al., 2018). Immune and metabolic disorders caused by uncontrolled exogenous substances entering the circulatory system as a result of gut leakiness may be the origin of DM.

### Immune disorder: The key to developing T1DM caused by gut microbiota dysbiosis and gut leakiness

Gut microbiota significantly influences the development of the immune system. Human and animal studies support a causal relationship between early microbial exposure and immune function development (Zhou et al., 2021). Defective immune response maturation is associated with T1DM progression later in life (Zhou et al., 2021). Moreover, the hygiene hypothesis posits that environmental improvements and antibiotic use lead to a reduction in microbiota diversity, possibly associated with a significant increase in allergic diseases and certain autoimmune diseases (Bach, 2018). Notably, the host’s genetic set-up of can also interact with the gut microbiota, leading to alterations in microbial composition, immune system activation, and T1DM susceptibility (Alkanani et al., 2015; Bonder et al., 2016).

It is suggested that gut leakiness possibly leads to uncontrol entry of external antigens (Di Tommaso et al., 2021), which could activate islet autoimmunity and directly damage pancreatic β cells (Sun et al., 2015; Bachem et al., 2019). Lipopolysaccharides (LPS) are a possible molecular link between the gut microbiota, inflammation, and T1DM (Bachem et al., 2019), an integral part of the outer membrane of Gram-negative bacterial species. The leakage of fatty acids and LPS can activate the toll-like receptor 4 (TLR4) (Velloso et al., 2015). Activation of TLR contributes to the maturation of dendritic cells and the recognition of pathogen-associated molecular patterns (Li et al., 2013). TLR4 is a member of patten-recognition receptors, and its activation helps activate pro-inflammatory signaling pathways, expressing and secreting cytokine while bacterial pathogens present (Shi et al., 2006; Cani et al., 2007). A case–control study proved that T1DM patients have higher circulating LPS levels than healthy individuals (Devaraj et al., 2009). In addition, these antigens may be absorbed by antigen-presenting cells (APCs) in the gut mucosa, which then activates the islet-reactive T cells that would be subsequently transported to pancreatic lymph nodes and islets to induce the damage of β cells in genetically predisposed individuals (Sorini et al., 2019). Some antigens may significantly be homologous to islet autoantigen, known as molecular mimicry, so they can directly induce the activation of pathogenic CD8+ T cells to promote DM development (Tai et al., 2016).

### Metabolites disorder: The key to developing T2DM induced by gut microbiota dysbiosis and gut leakiness

Like T1DM, gut microbiota dysbiosis in T2DM can increase serum LPS concentration to injure the intestinal barrier and change in mucosal immune response (Allcock et al., 2001). LPS and other antigens can activate TLR4 on immune cells and induce pro-inflammatory response and IR (Medzhitov, 2001; Medzhitov and Horng, 2009; Janssen and Kersten, 2017). Besides, gut leakiness leads to macrophage infiltration, which causes local inflammation by producing and activating serum IL-6, TNF-α, and other inflammatory cytokines (Ghosh et al., 2020). Gut leakiness may also introduce the gut microbiota and its metabolite into the blood systemic circulation and promote local and systemic immune responses (Sudo et al., 2002; Sumida et al., 2022). However, T2DM is traditionally characterized as a metabolic disease, so the role of gut microbiota metabolite is more remarkable in the development of T2DM. Gut microbiota produces bioactive metabolites, including short-chain fatty acids (SCFAs), ammonia, phenols, endotoxins, etc., through dietary macronutrients (Schroeder and Bäckhed, 2016). SCFAs, bile acids, indole derivatives, sulfur-containing amino acids, and vitamins are gut microbiota metabolisms that prevent DM, whereas Branched-chain amino acids (BCAAs), phenol, p-cresol, methane, amines and ammonia are gut microbiota metabolic that promote DM progression (Khan et al., 2014). Herein, we review the effect of several important metabolites in T2DM.

The short-chain fatty acids (SCFAs), such as acetate, propionate, and butyrate, are products of the metabolism of soluble fiber and amino acids by gut microbiota. Widely reported studies have demonstrated that SCFAs contribute to improved glucose homeostasis and metabolism in tissues such as the liver, adipose, and muscle (Canfora et al., 2015). Acetate can induce the browning of adipose tissue, and propionate stimulates the release of peptide YY (PYY) and glucagon-like peptide 1 (GLP-1) to reduce energy intake, whereas butyrate can reduce inflammation and reverse the decline in GLP-1 receptor expression in the liver (Sahuri-Arisoylu et al., 2005; Chambers et al., 2015; Jin et al., 2015; Zhou et al., 2017, 2018). In addition, two receptors for SCFAs, free fatty acid receptor 3 (GPR41) and free fatty acid receptor 2 (GPR43), have been identified to be directly implicated in T2DM development, which are expressed in enteroendocrine cells, intestinal epithelial cells, pancreatic islet cells, and other cells (Stoddart and Smith, 2008; Priyadarshini et al., 2018). Activation of GPR41 was reported to stimulate leptin secretion to regulate energy expenditure and long-term food intake (Xiong et al., 2004) and peptide YY (PYY) to increase satiety (Larraufie et al., 2018). In addition, activating the GPR41 in the sympathetic nervous system can stimulate energy expenditure and reduce the risk of T2DM (Kimura et al., 2011). GLP-1 will be increasingly released when GPR43 is activated, which enhances insulin secretion, suppresses glucagon production, and increases satiety (Tolhurst et al., 2012). Of note, GLP-1 might also improve endothelial cell function by changing the composition of gut microbiota (Chen et al., 2022). The GPR43 transgenic mouse showed improved metabolic parameters, such as reduced obesity, improved homeostasis, improved lean meat quality, and higher GLP-1 secretion (Bjursell et al., 2011; Tolhurst et al., 2012; Kimura et al., 2013). Moreover, it has been demonstrated that SCFAs can decrease inflammation in mucosal and chronic systemic, possibly because of their ability to inhibit pro-inflammatory cytokines like interleukin-6 (IL-6) and tumor necrosis factor α (TNF-α) (Roelofsen et al., 2010), induct anti-inflammatory cytokines (Säemann et al., 2000) and decrease the infiltration of inflammatory cells into adipose tissue (Meijer et al., 2010).

Primary bile acids are generated by the liver from cholesterol and released into the intestine by the gallbladder. The majority of these bile acids are reabsorbed in the intestinal tract, while a small amount reaches the lower intestine tract and is converted by the gut microbiota into secondary bile acid. Glucose homeostasis and intestinal fat absorption are both impacted by primary and secondary bile acids, which may significantly impact the pathophysiology of T2DM (Ahmad and Haeusler, 2019). Bile acids can lessen the amount of gluconeogenesis in the liver, enhance the production of glycogen, boost energy expenditure, stimulate the release of insulin, and reduce inflammation (Shapiro et al., 2018). These bile acids attach to and trigger nuclear hormone receptors like G-protein-coupled bile acid receptor-1 (TGR5) and nuclear hormone receptors, including the Pregnane X receptor (PXR) and Farnesoid X receptor (FXR). TGR5 is presented in digestive, immune, and adipose tissues. Intestinal TGR5 activation promotes insulin secretion and satiety by inducing the release of GLP-1 and PYY (Katsuma et al., 2005; Thomas et al., 2009; Kuhre et al., 2018). On the contrary, the knockout of TRG5 in myeloid-lineage cells and macrophages promoted IR and inflammation in adipose tissue (Perino et al., 2014). Moreover, the action of thyroid hormones may be promoted by bile acid-driven activation of TGR5 in adipose tissue, leading to an increase in energy expenditure (Watanabe et al., 2006). FXP is widely expressed in the liver, gut, kidney, and other tissues. The application of a synthetic FXR agonist was shown to significantly reduce plasma glucose, free fatty acids, triglycerides, cholesterol, and hepatic steatosis in DM mice (Zhang et al., 2006; Fang et al., 2015). Activation of PXR contributed to reducing bile acid production and increasing bile acid clearance to promote the absorption of lipid and fat-soluble vitamins (Staudinger et al., 2001). In addition, bile acids can stimulate GLP-1 secretion to regulate glucose metabolism and improve insulin sensitivity (Pathak et al., 2018; Shapiro et al., 2018).

Branched-chain amino acids (BCAAs) are essential amino acids produced by gut microbiota, including isoleucine, leucine, and valine. A case-cohort study supported that higher baseline BCAAs and their increase over 1 year were associated with a risk of T2DM (Ruiz-Canela et al., 2018). The obese mice model restored metabolic health while feeding them a reduced BCAAs diet, including improved insulin sensitivity and glucose tolerance, despite continuing to consume a high-sugar and high-fat diet (Cummings et al., 2018). However, a mendelian randomization analysis study suggested that the genetic risk score (GRS) for circulating BCAAs level was not linked to the homeostasis model assessment of insulin resistance (HOMA-IR), while the GRS for IR traits was significantly linked to increased circulating BCAAs level (Mahendran et al., 2017). This result indicates that it is not the increase of BCAAs that leads to IR, but IR that leads to the increase of BCAAs. The altered BCAAs levels are possibly due to reduced suppression of proteolysis caused by IR and reductions in BCAA catabolism in peripheral tissues caused by adiponectin signaling in T2DM (Lian et al., 2015; Giesbertz and Daniel, 2016).

In addition to the metabolites mentioned above, Trimethylamine (TMA), Indole derivatives, Imidazole propionate, etc., are associated with T2DM. TMA will be oxidized in the liver into Trimethylamine oxide (TMAO), and a case–control study reported that TMAO is positively associated with newly diagnosed T2DM (Shan et al., 2017). Indolepropionic acid is the product of the microbial metabolism of tryptophan, which is negatively linked to the risk of developing IR, low-grade inflammation, and T2DM (de Mello et al., 2017). Imidazole propionate is a microbial metabolite from histidine, which was demonstrated to be higher in T2DM patients compared with healthy individuals (Koh et al., 2018).

## Progress in microbial-based therapies in DM

With the growing understanding of the role of gut microbiota in DM, more and more researchers are trying to use microbial-based therapies to treat DM. These therapies directly or indirectly alter the composition of gut microbiota to function. Herein, we review the progress in microbial-based therapies to treat DM.

### Probiotics and prebiotics

Probiotics are referred to living microorganisms that are beneficial to the host’s health when administered in adequate amounts (Hill et al., 2014). Research on probiotics has pointed out their ability to enhance gut barrier function, regulate immunity, and competitively adhere to mucus and epithelial cells to benefit DM (Lomax and Calder, 2009; Ohland and Macnaughton, 2010). In animal models, oral administration of the probiotics increased the anti-inflammatory cytokines, such as TGF-β and IL-10 (Calcinaro et al., 2005; Sturm et al., 2005), decreased the pro-inflammatory cytokines like TNF-α, IL-6, and IL-1β (Mariño et al., 2017), and regulated the immune balance between Th1/Th2/ Th17/Treg cells (Lau et al., 2011; Jia et al., 2017). In addition, bone mineral density was positively correlated with the number of lactic acid bacteria and *Bifidobacterium* in DM patients (Hou et al., 2017).

Human and animal trials have demonstrated the potential of probiotics in preventing and treating DM (Que et al., 2021). It has been reported that the onset of DM in non-obese diabetics (NOD) was reduced by the early supplement of complex-probiotic-preparation VSL#3 (Calcinaro et al., 2005). Another research identified that early supplemental probiotic use reduced the risk of islet autoimmunity in HLA genotype T1DM high-risk children (Uusitalo et al., 2016), while another demonstrated a negative result (Savilahti et al., 2018). Early oral probiotic medication *Clostridium butyricum* was also observed to prevent NOD mice from developing DM (Jia et al., 2017). Combination of galactooligosaccharides, *Bifidobacterium breve strain Yakult*, and *Lacticaseibacillus paracasei strain Shirota* improved the amounts of total *lactobacilli* and *Bifidobacterium*, the proportions of *Bifidobacterium*, and the concentrations of acetate and butyrate in excrement in T2DM patients (Kanazawa et al., 2021).

Colonizing the host gut is an important process by which probiotics function. However, according to a systematic review, probiotics had no impact on the fecal microbiome composition in six of the seven studies that have been examined (Kristensen et al., 2016). Therefore, using prebiotics to promote the propagation of probiotics is another idea to regulate gut microbiota composition. Prebiotics are non-digestible dietary components that promote the host’s health by regulating the gut microbiota composition, especially by augmenting the abundance of *Bifidobacteria* and/or *Lactobacillus* (Jakaitis and Denning, 2014). Prebiotics improved glycemic control and decreased intestinal permeability in T1DM patients in a randomized placebo-controlled study, which enhanced insulin sensitivity (Ho et al., 2019). Human milk oligosaccharides (HMOs), the most common prebiotics that comprises multiple prebiotic oligosaccharides, are one of the most prevalent constituents of human milk (Knip and Honkanen, 2017). HMOs are believed to protect against autoimmune DM, possibly through selectively stimulating the growth of *Bifidobacteria* (Brown et al., 2011; de Goffau et al., 2013). In addition, HMOs also have microbially independent immunomodulatory properties, such as the induction of Treg cells (Lehmann et al., 2015) and the ability to maintain intestinal integrity (Aakko et al., 2017). Other prebiotics such as long-chain inulin-type fructosan and β-glucan-rich products have also been demonstrated to directly inhibit the progression of insulitis in NOD mice and reduce the incidence of DM (Chen et al., 2017; Gudi et al., 2019).

### Fecal microbiota transplantation

FMT refers to the transfer of healthy microbiomes to dysregulated receptors in the gut microbiota to restore the normal bacterial community (Vindigni and Surawicz, 2017). Notably, these healthy microbiomes can be not only allogeneic (allo-FMT) but also autologous (auto-FMT). Auto-FMT is often implemented by collecting someone’s microbiota when they are healthy and transplanting it back when their gut microbiota is out of whack. Participants with abdominal obesity or dyslipidemia can maintain losing weight and controlling blood glucose after accepting auto-FMT whose gut microbiota was collected during the weight-loss intervention phase (Rinott et al., 2021). Another recent randomized controlled trial discovered that auto-FMT prevented the decline in endogenous insulin secretion for 12 months after onset in patients with a recent diagnosis of T1DM, suggesting FMT can possibly prevent the ongoing β cells damage in T1DM patients (de Groot et al., 2021). Compared to auto-FMT, allo-FMT transplants healthy donors’ gut microbiota to receivers, which has been an important therapy in treating chronic diarrhea caused by *Clostridium difficile* infections (van Nood et al., 2013; Saha et al., 2021). Early-life FMT from MyD88-deficient mice has been shown to significantly delay the onset of T1DM in NOD mice (Peng et al., 2014). In addition, the development of T1DM was also significantly delayed by the non-selective transplantation of human gut microbiota into GF NOD mice (Neuman et al., 2019). On the contrary, the incidence of T1DM significantly increased when mice received antibiotics (Livanos et al., 2016). Of note, in newly diagnosed T1DM patients, auto-FMT delivered through duodenal tubes is more effective than allo-FMT in protecting β-cell function (de Groot et al., 2021).

In recent years, the use of FMT in the treatment of T2DM has also made great progress. A study with T2DM mice discovered that FMT can reduce hyperglycemia, improve IR, inhibit the level of chronic inflammation in pancreatic, and reduce β-cell apoptosis (Wang et al., 2019). Similarly, it has been demonstrated that the insulin sensitivity of patients with metabolic syndrome increased after receiving the transfer of gut microbiota from lean donors (Vrieze et al., 2012; Kootte et al., 2017). In addition, a randomized clinical trial indicated that butyrate-producing microbiota increased while transferring the microbiota from healthy lean donors to T2DM patients (Ng et al., 2022). Moreover, recent research began to try to combine FMT with dietary or lifestyle intervention to treat T2DM and got better results than single FMT (Ng et al., 2022; Su et al., 2022). It has been observed that FMT can increase the abundance of beneficial microbiota such as *Bifidobacterium* and decrease the level of *Sulfate-reducing bacteria*, *Desulfovibrio*, and *Bilophila* (Ng et al., 2022; Su et al., 2022). Similar to probiotics, FMT also has difficulty in colonizing the gut, but repeated FMTs can significantly increase the engraftment of lean-associated microbiota (Ng et al., 2022). Notably, FMT transfers not only the healthy microbiota to recipients but also compounds of potentially dangerous microbes (Walker, 2017; Hanssen et al., 2021).

### Dietary intervention

Diet and nutrition play the most important influence on how the gut microbiota and the host interact over a lifetime. The ingestion of nutrients influences the composition of microbial metabolism and serves as a substrate for it (Albenberg and Wu, 2014). Therefore, diet interventions are effective ways to alter the gut microbiota composition and influence the host’s health. The earliest human diet in life is breastmilk, and a meta-analysis elucidate that breastfed infant presents a stable and *Bifidobacteria*-dominating gut microbiota community, which is conducive to immune maturation (Uusitalo et al., 2016). A case–control study that found a lengthy breastfeeding time to be linked to a lower risk of T1DM suggests that exclusive and long-term breastfeeding is an independent protective factor for T1DM (Rosenbauer et al., 2008).

It has been reported that an animal-based diet reduced the abundance of metabolized plant polysaccharides *Firmicutes*, leading to a reduction in beneficial SCFAs (David et al., 2014), while high dietary fiber intake selectively promoted the growth of a group of SCFA-producing gut microbiota and increased HbA1c levels possibly by increasing the production of GLP-1 (Zhao et al., 2018). After 1 month of a vegan diet, obese patients with T2DM and/or hypertension presented a reduced F/B ratio and increased *Clostridium* and *Bacteroidetes fragile*, and they experienced significant reductions in HbA1c and triglyceride levels, weight loss, and improved fasting and postprandial glucose levels (Kim et al., 2013).In addition, high-fat diets regulate the composition of gut microbiota, mainly reducing the number of *Bifidobacterium* (Marietta et al., 2013). F/B ratio renewed while obese patients accepted carbohydrate-restricted or fat-restricted low-calorie diets (Cotillard et al., 2013; Bouter et al., 2017). Fiber-rich diet contributes to increasing *Prevotella*, whereas a protein-rich diet is associated with an increased abundance of *Bacteroides*. (Hartstra et al., 2015).

Concretely, green tea, caffeine, and omega-3 polyunsaturated fatty acids are beneficial for restoring the changed gut microbiota composition (Lau and Wong, 2018; Pascale et al., 2018). Consuming guar gum promoted T2DM patients to experience lipid-lowering effects (Li et al., 2021). Gluten intake promotes T1DM development by altering gut microbiota composition and immune response resulting in β -cell damage (Marietta et al., 2013). Zinc deficiency influences the inflammatory response and metabolic control (Xia et al., 2017), whereas Vitamin A deficiency increases the F/B ratio and decreases butyrate-producing gut microbiota level (Tian et al., 2018).

### Antidiabetic drugs

The gut microbiota interacts with various popular hypoglycemic drugs, such as metformin, liraglutide, acarbose, and thiazolidinedione (Gurung et al., 2020; Lee et al., 2021; Smits et al., 2021; Takewaki et al., 2021). The anti-hyperglycemic effect of metformin has been traditionally attributed to its direct action on the signaling process in liver cells, leading to lower hepatic gluconeogenesis. However, it has been reported that metformin can alleviate the reduction of butyrate-producing microbiota, and gut microbiota supports the therapeutic effects of metformin through SCFAs production (Forslund et al., 2015). Treating the newly diagnosed T2DM naively with metformin can increase the amount of the bile acid GUDCA, thus improving IR (Sun et al., 2018). A randomized controlled trial showed that metformin strongly affected the gut microbiota and germ-free mice that received a transfer of fecal samples from metformin-treated donors presented improved glucose tolerance (Wu et al., 2017). In a rodent model, the level of *Lactobacillus* in the upper small intestine was increased, and sodium-glucose cotransporter-1 (SGLT1) expression was restored, thus increasing glucose sensitivity after using metformin (Bauer et al., 2018).

## Conclusion and perspectives

In conclusion, the gut microbiota plays an important role in the occurrence and development of DM, and its composition in both T1DM and T2DM patients differs from that of healthy individuals. In general, opportunistic pathogens are usually reported increasing in DM patients, while probiotics are decreased. This review discusses the role of immune responses, inflammatory responses, metabolic disorders, and other aspects caused by gut microbiota dysbiosis in the pathogenesis of DM, enriching the content of intervention of DM through gut microbiota. This article also summarizes the progress of microbiological therapy in the prevention and treatment of DM, especially the current situation and application prospect of FMT in DM. Nowadays, there are a variety of drugs available to intervene in DM, but these drugs cannot sustainably and consistently prevent the progression of β-cell failure after the onset of DM. Therefore, it is of profound significance to intervene in DM through gut microbiota. In the future, we will verify the effectiveness of gut microbiota intervention in DM through clinical trials, and further, explore the advantages of gut microbiota intervention in DM.

## Author contributions

JY and ZW were responsible for the design and manuscript writing. JY, ZW, and YZ were responsible for editing the structure of the article, obtaining the documents, and further arranging the manuscripts. YS and WL were responsible for the supervision, review, and final editing of the manuscript. All authors contributed to the article and approved the submitted version.

## Conflict of interest

The authors declare that the research was conducted in the absence of any commercial or financial relationships that could be construed as a potential conflict of interest.

## Publisher’s note

All claims expressed in this article are solely those of the authors and do not necessarily represent those of their affiliated organizations, or those of the publisher, the editors and the reviewers. Any product that may be evaluated in this article, or claim that may be made by its manufacturer, is not guaranteed or endorsed by the publisher.

## References

[ref1] Aakko J. Kumar H. Rautava S. Wise A. Autran C. Bode L.. (2017). Human milk oligosaccharide categories define the microbiota composition in human colostrum. Benefic. Microbes 8, 563–567. doi: 10.3920/BM2016.0185, PMID: 28726512

[ref2] Ahmad T. R. Haeusler R. A. (2019). Bile acids in glucose metabolism and insulin signalling-mechanisms and research needs. Nat. Rev. Endocrinol. 15, 701–712. doi: 10.1038/s41574-019-0266-7, PMID: 31616073 PMC6918475

[ref3] Albenberg L. G. Wu G. D. (2014). Diet and the intestinal microbiome: associations, functions, and implications for health and disease. Gastroenterology 146, 1564–1572. doi: 10.1053/j.gastro.2014.01.058, PMID: 24503132 PMC4216184

[ref4] Alkanani A. K. Hara N. Gottlieb P. A. Ir D. Robertson C. E. Wagner B. D.. (2015). Alterations in intestinal microbiota correlate with susceptibility to type 1 diabetes. Diabetes 64, 3510–3520. doi: 10.2337/db14-1847, PMID: 26068542 PMC4587635

[ref5] Allcock G. H. Allegra M. Flower R. J. Perretti M. (2001). Neutrophil accumulation induced by bacterial lipopolysaccharide: effects of dexamethasone and annexin 1. Clin. Exp. Immunol. 123, 62–67. doi: 10.1046/j.1365-2249.2001.01370.x, PMID: 11167999 PMC1905950

[ref6] Allin K. H. Tremaroli V. Caesar R. Jensen B. A. H. Damgaard M. T. F. Bahl M. I.. (2018). Aberrant intestinal microbiota in individuals with prediabetes. Diabetologia 61, 810–820. doi: 10.1007/s00125-018-4550-1, PMID: 29379988 PMC6448993

[ref7] Amabebe E. Robert F. O. Agbalalah T. Orubu E. S. F. (2020). Microbial dysbiosis-induced obesity: role of gut microbiota in homoeostasis of energy metabolism. Br. J. Nutr. 123, 1127–1137. doi: 10.1017/S0007114520000380, PMID: 32008579

[ref8] Arboleya S. Watkins C. Stanton C. Ross R. P. (2016). Gut Bifidobacteria populations in human health and aging. Front. Microbiol. 7:1204. doi: 10.3389/fmicb.2016.01204 27594848 PMC4990546

[ref9] Atkinson M. A. Eisenbarth G. S. Michels A. W. (2014). Type 1 diabetes. Lancet 383, 69–82. doi: 10.1016/S0140-6736(13)60591-7, PMID: 23890997 PMC4380133

[ref10] Bach J. F. (2018). The hygiene hypothesis in autoimmunity: the role of pathogens and commensals. Nat. Rev. Immunol. 18, 105–120. doi: 10.1038/nri.2017.111, PMID: 29034905

[ref11] Bachem A. Makhlouf C. Binger K. J. de Souza D. P. Tull D. Hochheiser K.. (2019). Microbiota-derived short-chain fatty acids promote the memory potential of antigen-activated CD8(+) T cells. Immunity 51, 285–97.e5. doi: 10.1016/j.immuni.2019.06.002, PMID: 31272808

[ref12] Bauer P. V. Duca F. A. Waise T. M. Z. Rasmussen B. A. Abraham M. A. Dranse H. J.. (2018). Metformin alters upper small intestinal microbiota that impact a glucose-SGLT1-sensing Glucoregulatory pathway. Cell Metab. 27:e5, 101–117.e5. doi: 10.1016/j.cmet.2017.09.019, PMID: 29056513

[ref13] Bibbò S. Dore M. P. Pes G. M. Delitala G. Delitala A. P. (2017). Is there a role for gut microbiota in type 1 diabetes pathogenesis? Ann. Med. 49, 11–22. doi: 10.1080/07853890.2016.1222449, PMID: 27499366

[ref14] Bjursell M. Admyre T. Göransson M. Marley A. E. Smith D. M. Oscarsson J.. (2011). Improved glucose control and reduced body fat mass in free fatty acid receptor 2-deficient mice fed a high-fat diet. Am. J. Physiol. Endocrinol. Metab. 300, E211–E220. doi: 10.1152/ajpendo.00229.2010, PMID: 20959533

[ref15] Bonder M. J. Kurilshikov A. Tigchelaar E. F. Mujagic Z. Imhann F. Vila A. V.. (2016). The effect of host genetics on the gut microbiome. Nat. Genet. 48, 1407–1412. doi: 10.1038/ng.3663 27694959

[ref16] Bouter K. E. van Raalte D. H. Groen A. K. Nieuwdorp M. (2017). Role of the gut microbiome in the pathogenesis of obesity and obesity-related metabolic dysfunction. Gastroenterology 152, 1671–1678. doi: 10.1053/j.gastro.2016.12.048, PMID: 28192102

[ref17] Brown C. T. Davis-Richardson A. G. Giongo A. Gano K. A. Crabb D. B. Mukherjee N.. (2011). Gut microbiome metagenomics analysis suggests a functional model for the development of autoimmunity for type 1 diabetes. PLoS One 6:e25792. doi: 10.1371/journal.pone.0025792, PMID: 22043294 PMC3197175

[ref18] Calcinaro F. Dionisi S. Marinaro M. Candeloro P. Bonato V. Marzotti S.. (2005). Oral probiotic administration induces interleukin-10 production and prevents spontaneous autoimmune diabetes in the non-obese diabetic mouse. Diabetologia 48, 1565–1575. doi: 10.1007/s00125-005-1831-2, PMID: 15986236

[ref19] Canfora E. E. Jocken J. W. Blaak E. E. (2015). Short-chain fatty acids in control of body weight and insulin sensitivity. Nat. Rev. Endocrinol. 11, 577–591. doi: 10.1038/nrendo.2015.128 26260141

[ref20] Cani P. D. Amar J. Iglesias M. A. Poggi M. Knauf C. Bastelica D.. (2007). Metabolic endotoxemia initiates obesity and insulin resistance. Diabetes 56, 1761–1772. doi: 10.2337/db06-1491, PMID: 17456850

[ref21] Chambers E. S. Viardot A. Psichas A. Morrison D. J. Murphy K. G. Zac-Varghese S. E.. (2015). Effects of targeted delivery of propionate to the human colon on appetite regulation, body weight maintenance and adiposity in overweight adults. Gut 64, 1744–1754. doi: 10.1136/gutjnl-2014-307913, PMID: 25500202 PMC4680171

[ref22] Chen K. Chen H. Faas M. M. de Haan B. J. Li J. Xiao P.. (2017). Specific inulin-type fructan fibers protect against autoimmune diabetes by modulating gut immunity, barrier function, and microbiota homeostasis. Mol. Nutr. Food Res. 61. doi: 10.1002/mnfr.201601006, PMID: 28218451

[ref23] Chen F. He L. Li J. Yang S. Zhang B. Zhu D.. (2022). Polyethylene glycol Loxenatide injection (GLP-1) protects vascular endothelial cell function in middle-aged and elderly patients with type 2 diabetes by regulating gut microbiota. Front. Mol. Biosci. 9:879294. doi: 10.3389/fmolb.2022.879294, PMID: 35782875 PMC9240776

[ref24] Cinek O. Kramna L. Mazankova K. Odeh R. Alassaf A. Ibekwe M. U.. (2018). The bacteriome at the onset of type 1 diabetes: a study from four geographically distant African and Asian countries. Diabetes Res. Clin. Pract. 144, 51–62. doi: 10.1016/j.diabres.2018.08.010, PMID: 30121305

[ref25] Classification and Diagnosis of Diabetes (2022). Standards of medical Care in Diabetes-2022. Diabetes Care 45, S17–s38. doi: 10.2337/dc22-S002 34964875

[ref26] Cotillard A. Kennedy S. P. Kong L. C. Prifti E. Pons N. Le Chatelier E.. (2013). Dietary intervention impact on gut microbial gene richness. Nature 500, 585–588. doi: 10.1038/nature12480, PMID: 23985875

[ref27] Cummings N. E. Williams E. M. Kasza I. Konon E. N. Schaid M. D. Schmidt B. A.. (2018). Restoration of metabolic health by decreased consumption of branched-chain amino acids. J. Physiol. 596, 623–645. doi: 10.1113/JP275075, PMID: 29266268 PMC5813603

[ref28] Danaei G. Finucane M. M. Lu Y. Singh G. M. Cowan M. J. Paciorek C. J.. (2011). National, regional, and global trends in fasting plasma glucose and diabetes prevalence since 1980: systematic analysis of health examination surveys and epidemiological studies with 370 country-years and 2·7 million participants. Lancet 378, 31–40. doi: 10.1016/S0140-6736(11)60679-X, PMID: 21705069

[ref29] Dave M. Higgins P. D. Middha S. Rioux K. P. (2012). The human gut microbiome: current knowledge, challenges, and future directions. Transl. Res. 160, 246–257. doi: 10.1016/j.trsl.2012.05.003, PMID: 22683238

[ref30] David L. A. Maurice C. F. Carmody R. N. Gootenberg D. B. Button J. E. Wolfe B. E.. (2014). Diet rapidly and reproducibly alters the human gut microbiome. Nature 505, 559–563. doi: 10.1038/nature12820, PMID: 24336217 PMC3957428

[ref31] de Goffau M. C. Luopajärvi K. Knip M. Ilonen J. Ruohtula T. Härkönen T.. (2013). Fecal microbiota composition differs between children with β-cell autoimmunity and those without. Diabetes 62, 1238–1244. doi: 10.2337/db12-0526, PMID: 23274889 PMC3609581

[ref32] de Groot P. Nikolic T. Pellegrini S. Sordi V. Imangaliyev S. Rampanelli E.. (2021). Faecal microbiota transplantation halts progression of human new-onset type 1 diabetes in a randomised controlled trial. Gut 70, 92–105. doi: 10.1136/gutjnl-2020-322630, PMID: 33106354 PMC7788262

[ref33] de Mello V. D. Paananen J. Lindström J. Lankinen M. A. Shi L. Kuusisto J.. (2017). Indolepropionic acid and novel lipid metabolites are associated with a lower risk of type 2 diabetes in the Finnish diabetes prevention study. Sci. Rep. 7:46337. doi: 10.1038/srep46337, PMID: 28397877 PMC5387722

[ref34] Devaraj S. Dasu M. R. Park S. H. Jialal I. (2009). Increased levels of ligands of toll-like receptors 2 and 4 in type 1 diabetes. Diabetologia 52, 1665–1668. doi: 10.1007/s00125-009-1394-8, PMID: 19455302 PMC2709882

[ref35] Di Tommaso N. Gasbarrini A. Ponziani F. R. (2021). Intestinal barrier in human health and disease. Int. J. Environ. Res. Public Health 18. doi: 10.3390/ijerph182312836, PMID: 34886561 PMC8657205

[ref36] Durazzo M. Ferro A. Gruden G. (2019). Gastrointestinal microbiota and type 1 diabetes mellitus: the state of art. J. Clin. Med. 8. doi: 10.3390/jcm8111843, PMID: 31684011 PMC6912450

[ref37] Fan Y. Pedersen O. (2021). Gut microbiota in human metabolic health and disease. Nat. Rev. Microbiol. 19, 55–71. doi: 10.1038/s41579-020-0433-9 32887946

[ref38] Fang S. Suh J. M. Reilly S. M. Yu E. Osborn O. Lackey D.. (2015). Intestinal FXR agonism promotes adipose tissue browning and reduces obesity and insulin resistance. Nat. Med. 21, 159–165. doi: 10.1038/nm.3760, PMID: 25559344 PMC4320010

[ref39] Forslund K. Hildebrand F. Nielsen T. Falony G. Le Chatelier E. Sunagawa S.. (2015). Disentangling type 2 diabetes and metformin treatment signatures in the human gut microbiota. Nature 528, 262–266. doi: 10.1038/nature15766, PMID: 26633628 PMC4681099

[ref40] Ghosh S. S. Wang J. Yannie P. J. Ghosh S. (2020). Intestinal barrier dysfunction, LPS translocation, and disease development. Journal of the endocrine. Society 4:bvz039. doi: 10.1210/jendso/bvz039 PMC7033038 32099951

[ref41] Giesbertz P. Daniel H. (2016). Branched-chain amino acids as biomarkers in diabetes. Curr. Opin. Clin. Nutr. Metab. Care 19, 48–54. doi: 10.1097/MCO.0000000000000235 26485337

[ref42] Gudi R. Perez N. Johnson B. M. Sofi M. H. Brown R. Quan S.. (2019). Complex dietary polysaccharide modulates gut immune function and microbiota, and promotes protection from autoimmune diabetes. Immunology 157, 70–85. doi: 10.1111/imm.13048, PMID: 30712258 PMC6459770

[ref43] Guigoz Y. Doré J. Schiffrin E. J. (2008). The inflammatory status of old age can be nurtured from the intestinal environment. Curr. Opin. Clin. Nutr. Metab. Care 11, 13–20. doi: 10.1097/MCO.0b013e3282f2bfdf, PMID: 18090652

[ref44] Gurung M. Li Z. You H. Rodrigues R. Jump D. B. Morgun A.. (2020). Role of gut microbiota in type 2 diabetes pathophysiology. EBioMedicine 51:102590. doi: 10.1016/j.ebiom.2019.11.051, PMID: 31901868 PMC6948163

[ref45] Hague A. Butt A. J. Paraskeva C. (1996). The role of butyrate in human colonic epithelial cells: an energy source or inducer of differentiation and apoptosis? Proc. Nutr. Soc. 55, 937–943. doi: 10.1079/PNS19960090 9004335

[ref46] Han H. Li Y. Fang J. Liu G. Yin J. Li T.. (2018). Gut microbiota and type 1 diabetes. Int. J. Mol. Sci. 19. doi: 10.3390/ijms19040995, PMID: 29584630 PMC5979537

[ref47] Hanssen N. M. J. de Vos W. M. Nieuwdorp M. (2021). Fecal microbiota transplantation in human metabolic diseases: from a murky past to a bright future? Cell Metab. 33, 1098–1110. doi: 10.1016/j.cmet.2021.05.005, PMID: 34077717

[ref48] Hartstra A. V. Bouter K. E. Bäckhed F. Nieuwdorp M. (2015). Insights into the role of the microbiome in obesity and type 2 diabetes. Diabetes Care 38, 159–165. doi: 10.2337/dc14-0769, PMID: 25538312

[ref49] Hasani A. Ebrahimzadeh S. Hemmati F. Khabbaz A. Hasani A. Gholizadeh P. (2021). The role of Akkermansia muciniphila in obesity, diabetes, and atherosclerosis. J. Med. Microbiol. 70. doi: 10.1099/jmm.0.001435, PMID: 34623232

[ref50] Hill C. Guarner F. Reid G. Gibson G. R. Merenstein D. J. Pot B.. (2014). Expert consensus document: The international scientific Association for Probiotics and Prebiotics consensus statement on the scope and appropriate use of the term probiotic. Nat. Rev. Gastroenterol. Hepatol. 11, 506–514. doi: 10.1038/nrgastro.2014.66, PMID: 24912386

[ref51] Ho J. Nicolucci A. C. Virtanen H. Schick A. Meddings J. Reimer R. A.. (2019). Effect of prebiotic on microbiota, intestinal permeability, and glycemic control in children with type 1 diabetes. J. Clin. Endocrinol. Metab. 104, 4427–4440. doi: 10.1210/jc.2019-00481, PMID: 31188437

[ref52] Hooper L. V. Midtvedt T. Gordon J. I. (2002). How host-microbial interactions shape the nutrient environment of the mammalian intestine. Annu. Rev. Nutr. 22, 283–307. doi: 10.1146/annurev.nutr.22.011602.092259 12055347

[ref53] Hou K. J. Lin C. J. Chen C. Wu B. T. Zhu D. Zhong W. J.. (2017). Association of probiotics and bone mineral density in Chinese patients with type 2 diabetes. Biomed. Res. 28, 129–133.

[ref54] Hou K. Wu Z. X. Chen X. Y. Wang J. Q. Zhang D. Xiao C.. (2022a). Microbiota in health and diseases. Signal Transduct. Target. Ther. 7:135. doi: 10.1038/s41392-022-00974-4, PMID: 35461318 PMC9034083

[ref55] Hou K. Zhang S. Wu Z. Zhu D. Chen F. Lei Z. N.. (2022b). Reconstruction of intestinal microecology of type 2 diabetes by fecal microbiota transplantation: why and how. Bosn. J. Basic Med. Sci. 22, 315–325. doi: 10.17305/bjbms.2021.6323, PMID: 34761734 PMC9162745

[ref56] Isolauri E. (2012). Development of healthy gut microbiota early in life. J. Paediatr. Child Health 48, 1–6. doi: 10.1111/j.1440-1754.2012.02489.x 22681492

[ref57] Jakaitis B. M. Denning P. W. (2014). Human breast milk and the gastrointestinal innate immune system. Clin. Perinatol. 41, 423–435. doi: 10.1016/j.clp.2014.02.011, PMID: 24873841 PMC4414019

[ref58] Jandhyala S. M. Talukdar R. Subramanyam C. Vuyyuru H. Sasikala M. Nageshwar R. D. (2015). Role of the normal gut microbiota. World J. Gastroenterol. 21, 8787–8803. doi: 10.3748/wjg.v21.i29.8787, PMID: 26269668 PMC4528021

[ref59] Janssen A. W. Kersten S. (2017). Potential mediators linking gut bacteria to metabolic health: a critical view. J. Physiol. 595, 477–487. doi: 10.1113/JP272476, PMID: 27418465 PMC5233664

[ref60] Jia L. Shan K. Pan L. L. Feng N. Lv Z. Sun Y.. (2017). Clostridium butyricum CGMCC0313.1 protects against autoimmune diabetes by modulating intestinal immune homeostasis and inducing pancreatic regulatory T cells. Front. Immunol. 8:1345. doi: 10.3389/fimmu.2017.01345, PMID: 29097999 PMC5654235

[ref61] Jin C. J. Sellmann C. Engstler A. J. Ziegenhardt D. Bergheim I. (2015). Supplementation of sodium butyrate protects mice from the development of non-alcoholic steatohepatitis (NASH). Br. J. Nutr. 114, 1745–1755. doi: 10.1017/S0007114515003621, PMID: 26450277

[ref62] Kahn S. E. Cooper M. E. Del Prato S. (2014). Pathophysiology and treatment of type 2 diabetes: perspectives on the past, present, and future. Lancet 383, 1068–1083. doi: 10.1016/S0140-6736(13)62154-6, PMID: 24315620 PMC4226760

[ref63] Kanazawa A. Aida M. Yoshida Y. Kaga H. Katahira T. Suzuki L.. (2021). Effects of synbiotic supplementation on chronic inflammation and the gut microbiota in obese patients with type 2 diabetes mellitus: a randomized controlled study. Nutrients 13. doi: 10.3390/nu13020558, PMID: 33567701 PMC7914668

[ref64] Karlsson F. H. Tremaroli V. Nookaew I. Bergström G. Behre C. J. Fagerberg B.. (2013). Gut metagenome in European women with normal, impaired and diabetic glucose control. Nature 498, 99–103. doi: 10.1038/nature12198, PMID: 23719380

[ref65] Katsuma S. Hirasawa A. Tsujimoto G. (2005). Bile acids promote glucagon-like peptide-1 secretion through TGR5 in a murine enteroendocrine cell line STC-1. Biochem. Biophys. Res. Commun. 329, 386–390. doi: 10.1016/j.bbrc.2005.01.139, PMID: 15721318

[ref66] Kayama H. Okumura R. Takeda K. (2020). Interaction between the microbiota, epithelia, and immune cells in the intestine. Annu. Rev. Immunol. 38, 23–48. doi: 10.1146/annurev-immunol-070119-115104, PMID: 32340570

[ref67] Khan M. T. Nieuwdorp M. Bäckhed F. (2014). Microbial modulation of insulin sensitivity. Cell Metab. 20, 753–760. doi: 10.1016/j.cmet.2014.07.006 25176147

[ref68] Kim M. S. Hwang S. S. Park E. J. Bae J. W. (2013). Strict vegetarian diet improves the risk factors associated with metabolic diseases by modulating gut microbiota and reducing intestinal inflammation. Environ. Microbiol. Rep. 5, 765–775. doi: 10.1111/1758-2229.12079, PMID: 24115628

[ref69] Kimura I. Inoue D. Maeda T. Hara T. Ichimura A. Miyauchi S.. (2011). Short-chain fatty acids and ketones directly regulate sympathetic nervous system via G protein-coupled receptor 41 (GPR41). Proc. Natl. Acad. Sci. U. S. A. 108, 8030–8035. doi: 10.1073/pnas.1016088108, PMID: 21518883 PMC3093469

[ref70] Kimura I. Ozawa K. Inoue D. Imamura T. Kimura K. Maeda T.. (2013). The gut microbiota suppresses insulin-mediated fat accumulation via the short-chain fatty acid receptor GPR43. Nat. Commun. 4:1829. doi: 10.1038/ncomms2852, PMID: 23652017 PMC3674247

[ref71] Knip M. Honkanen J. (2017). Modulation of type 1 diabetes risk by the intestinal microbiome. Curr. Diab. Rep. 17:105. doi: 10.1007/s11892-017-0933-9, PMID: 28942491

[ref72] Koh A. Molinaro A. Ståhlman M. Khan M. T. Schmidt C. Mannerås-Holm L.. (2018). Microbially produced imidazole propionate impairs insulin signaling through mTORC1. Cells 175, 947–61.e17. doi: 10.1016/j.cell.2018.09.055, PMID: 30401435

[ref73] Kootte R. S. Levin E. Salojärvi J. Smits L. P. Hartstra A. V. Udayappan S. D.. (2017). Improvement of insulin sensitivity after lean donor feces in metabolic syndrome is driven by baseline intestinal microbiota composition. Cell Metab. 26, 611–9.e6. doi: 10.1016/j.cmet.2017.09.008, PMID: 28978426

[ref74] Kostic A. D. Gevers D. Siljander H. Vatanen T. Hyötyläinen T. Hämäläinen A. M.. (2015). The dynamics of the human infant gut microbiome in development and in progression toward type 1 diabetes. Cell Host Microbe 17, 260–273. doi: 10.1016/j.chom.2015.01.001, PMID: 25662751 PMC4689191

[ref75] Kristensen N. B. Bryrup T. Allin K. H. Nielsen T. Hansen T. H. Pedersen O. (2016). Alterations in fecal microbiota composition by probiotic supplementation in healthy adults: a systematic review of randomized controlled trials. Genome Med. 8:52. doi: 10.1186/s13073-016-0300-5, PMID: 27159972 PMC4862129

[ref76] Kuhre R. E. Wewer Albrechtsen N. J. Larsen O. Jepsen S. L. Balk-Møller E. Andersen D. B.. (2018). Bile acids are important direct and indirect regulators of the secretion of appetite and metabolism-regulating hormones from the gut and pancreas. Mol. Metab. 11, 84–95. doi: 10.1016/j.molmet.2018.03.007, PMID: 29656109 PMC6001409

[ref77] Landman C. Quévrain E. (2016). Gut microbiota: description, role and pathophysiologic implications. La Revue de Med. Int. 37, 418–423. doi: 10.1016/j.revmed.2015.12.012, PMID: 26749318

[ref78] Larraufie P. Martin-Gallausiaux C. Lapaque N. Dore J. Gribble F. M. Reimann F.. (2018). SCFAs strongly stimulate PYY production in human enteroendocrine cells. Sci. Rep. 8:74. doi: 10.1038/s41598-017-18259-0, PMID: 29311617 PMC5758799

[ref79] Larsen N. Vogensen F. K. van den Berg F. W. Nielsen D. S. Andreasen A. S. Pedersen B. K.. (2010). Gut microbiota in human adults with type 2 diabetes differs from non-diabetic adults. PLoS One 5:e9085. doi: 10.1371/journal.pone.0009085, PMID: 20140211 PMC2816710

[ref80] Lau K. Benitez P. Ardissone A. Wilson T. D. Collins E. L. Lorca G.. (2011). Inhibition of type 1 diabetes correlated to a lactobacillus johnsonii N6.2-mediated Th17 bias. J. Immunol. 186, 3538–3546. doi: 10.4049/jimmunol.1001864, PMID: 21317395

[ref81] Lau L. H. S. Wong S. H. (2018). Microbiota, obesity and NAFLD. Adv. Exp. Med. Biol. 1061, 111–125. doi: 10.1007/978-981-10-8684-7_9 29956210

[ref82] Lee Y. Kim A. H. Kim E. Lee S. Yu K. S. Jang I. J.. (2021). Changes in the gut microbiome influence the hypoglycemic effect of metformin through the altered metabolism of branched-chain and nonessential amino acids. Diabetes Res. Clin. Pract. 178:108985. doi: 10.1016/j.diabres.2021.108985, PMID: 34329692

[ref83] Lehmann S. Hiller J. van Bergenhenegouwen J. Knippels L. M. Garssen J. Traidl-Hoffmann C. (2015). *In vitro* evidence for immune-modulatory properties of non-digestible oligosaccharides: direct effect on human monocyte derived dendritic cells. PLoS One 10:e0132304. doi: 10.1371/journal.pone.0132304, PMID: 26148091 PMC4493044

[ref84] Leiva-Gea I. Sánchez-Alcoholado L. Martín-Tejedor B. Castellano-Castillo D. Moreno-Indias I. Urda-Cardona A.. (2018). Gut microbiota differs in composition and functionality between children with type 1 diabetes and MODY2 and healthy control subjects: a case-control study. Diabetes Care 41, 2385–2395. doi: 10.2337/dc18-0253, PMID: 30224347

[ref85] Li J. Chen R. Chen Y. Zhu D. Wu Z. Chen F.. (2021). The effect of guar gum consumption on the lipid profile in type 2 diabetes mellitus: a systematic review and meta-analysis of randomized controlled trials. Crit. Rev. Food Sci. Nutr. 1–10. doi: 10.1080/10408398.2021.1981228 34558350

[ref86] Li W. Ma Z. S. (2020). FBA ecological guild: trio of firmicutes-bacteroidetes alliance against actinobacteria in human oral microbiome. Sci. Rep. 10:287. doi: 10.1038/s41598-019-56561-1, PMID: 31937838 PMC6959321

[ref87] Li J. Wang X. Zhang F. Yin H. (2013). Toll-like receptors as therapeutic targets for autoimmune connective tissue diseases. Pharmacol. Ther. 138, 441–451. doi: 10.1016/j.pharmthera.2013.03.003, PMID: 23531543 PMC3686650

[ref88] Lian K. Du C. Liu Y. Zhu D. Yan W. Zhang H.. (2015). Impaired adiponectin signaling contributes to disturbed catabolism of branched-chain amino acids in diabetic mice. Diabetes 64, 49–59. doi: 10.2337/db14-0312, PMID: 25071024

[ref89] Livanos A. E. Greiner T. U. Vangay P. Pathmasiri W. Stewart D. McRitchie S.. (2016). Antibiotic-mediated gut microbiome perturbation accelerates development of type 1 diabetes in mice. Nat. Microbiol. 1:16140. doi: 10.1038/nmicrobiol.2016.140, PMID: 27782139 PMC5808443

[ref90] Lomax A. R. Calder P. C. (2009). Probiotics, immune function, infection and inflammation: a review of the evidence from studies conducted in humans. Curr. Pharm. Des. 15, 1428–1518. doi: 10.2174/138161209788168155, PMID: 19442167

[ref91] Mahendran Y. Jonsson A. Have C. T. Allin K. H. Witte D. R. Jørgensen M. E.. (2017). Genetic evidence of a causal effect of insulin resistance on branched-chain amino acid levels. Diabetologia 60, 873–878. doi: 10.1007/s00125-017-4222-6, PMID: 28184960

[ref92] Marietta E. V. Gomez A. M. Yeoman C. Tilahun A. Y. Clark C. R. Luckey D. H.. (2013). Low incidence of spontaneous type 1 diabetes in non-obese diabetic mice raised on gluten-free diets is associated with changes in the intestinal microbiome. PLoS One 8:e78687. doi: 10.1371/journal.pone.0078687, PMID: 24236037 PMC3827256

[ref93] Mariño E. Richards J. L. McLeod K. H. Stanley D. Yap Y. A. Knight J.. (2017). Gut microbial metabolites limit the frequency of autoimmune T cells and protect against type 1 diabetes. Nat. Immunol. 18, 552–562. doi: 10.1038/ni.3713, PMID: 28346408

[ref94] Medzhitov R. (2001). Toll-like receptors and innate immunity. Nat. Rev. Immunol. 1, 135–145. doi: 10.1038/35100529 11905821

[ref95] Medzhitov R. Horng T. (2009). Transcriptional control of the inflammatory response. Nat. Rev. Immunol. 9, 692–703. doi: 10.1038/nri2634 19859064

[ref96] Meijer K. de Vos P. Priebe M. G. (2010). Butyrate and other short-chain fatty acids as modulators of immunity: what relevance for health? Curr. Opin. Clin. Nutr. Metab. Care 13, 715–721. doi: 10.1097/MCO.0b013e32833eebe5, PMID: 20823773

[ref97] Mokhtari P. Metos J. Anandh Babu P. V. (2021). Impact of type 1 diabetes on the composition and functional potential of gut microbiome in children and adolescents: possible mechanisms, current knowledge, and challenges. Gut Microbes 13, 1–18. doi: 10.1080/19490976.2021.1926841, PMID: 34101547 PMC8205092

[ref98] Mu Q. Kirby J. Reilly C. M. Luo X. M. (2017). Leaky gut as a danger signal for autoimmune diseases. Front. Immunol. 8:598. doi: 10.3389/fimmu.2017.00598, PMID: 28588585 PMC5440529

[ref99] Murri M. Leiva I. Gomez-Zumaquero J. M. Tinahones F. J. Cardona F. Soriguer F.. (2013). Gut microbiota in children with type 1 diabetes differs from that in healthy children: a case-control study. BMC Med. 11:46. doi: 10.1186/1741-7015-11-46, PMID: 23433344 PMC3621820

[ref100] Natividad J. M. Petit V. Huang X. de Palma G. Jury J. Sanz Y.. (2012). Commensal and probiotic bacteria influence intestinal barrier function and susceptibility to colitis in nod 1−/−; nod 2−/− mice. Inflamm. Bowel Dis. 18, 1434–1446. doi: 10.1002/ibd.22848, PMID: 22162005

[ref101] Neuman V. Cinek O. Funda D. P. Hudcovic T. Golias J. Kramna L.. (2019). Human gut microbiota transferred to germ-free NOD mice modulate the progression towards type 1 diabetes regardless of the pace of beta cell function loss in the donor. Diabetologia 62, 1291–1296. doi: 10.1007/s00125-019-4869-2, PMID: 31025045

[ref102] Ng S. C. Xu Z. Mak J. W. Y. Yang K. Liu Q. Zuo T.. (2022). Microbiota engraftment after faecal microbiota transplantation in obese subjects with type 2 diabetes: a 24-week, double-blind, randomised controlled trial. Gut 71, 716–723. doi: 10.1136/gutjnl-2020-323617, PMID: 33785557

[ref103] Ohland C. L. Macnaughton W. K. (2010). Probiotic bacteria and intestinal epithelial barrier function. Am. J. Physiol. Gastrointest. Liver Physiol. 298, G807–G819. doi: 10.1152/ajpgi.00243.2009 20299599

[ref104] Pascale A. Marchesi N. Marelli C. Coppola A. Luzi L. Govoni S.. (2018). Microbiota and metabolic diseases. Endocrine 61, 357–371. doi: 10.1007/s12020-018-1605-5 29721802

[ref105] Pathak P. Xie C. Nichols R. G. Ferrell J. M. Boehme S. Krausz K. W.. (2018). Intestine farnesoid X receptor agonist and the gut microbiota activate G-protein bile acid receptor-1 signaling to improve metabolism. Hepatology 68, 1574–1588. doi: 10.1002/hep.29857, PMID: 29486523 PMC6111007

[ref106] Pellegrini S. Sordi V. Bolla A. M. Saita D. Ferrarese R. Canducci F.. (2017). Duodenal mucosa of patients with type 1 diabetes shows distinctive inflammatory profile and microbiota. J. Clin. Endocrinol. Metab. 102, 1468–1477. doi: 10.1210/jc.2016-3222, PMID: 28324102

[ref107] Peng L. He Z. Chen W. Holzman I. R. Lin J. (2007). Effects of butyrate on intestinal barrier function in a Caco-2 cell monolayer model of intestinal barrier. Pediatr. Res. 61, 37–41. doi: 10.1203/01.pdr.0000250014.92242.f3, PMID: 17211138

[ref108] Peng L. Li Z. R. Green R. S. Holzman I. R. Lin J. (2009). Butyrate enhances the intestinal barrier by facilitating tight junction assembly via activation of AMP-activated protein kinase in Caco-2 cell monolayers. J. Nutr. 139, 1619–1625. doi: 10.3945/jn.109.104638, PMID: 19625695 PMC2728689

[ref109] Peng J. Narasimhan S. Marchesi J. R. Benson A. Wong F. S. Wen L. (2014). Long term effect of gut microbiota transfer on diabetes development. J. Autoimmun. 53, 85–94. doi: 10.1016/j.jaut.2014.03.005, PMID: 24767831 PMC4361177

[ref110] Perino A. Pols T. W. Nomura M. Stein S. Pellicciari R. Schoonjans K. (2014). TGR5 reduces macrophage migration through mTOR-induced C/EBPβ differential translation. J. Clin. Invest. 124, 5424–5436. doi: 10.1172/JCI76289, PMID: 25365223 PMC4348975

[ref111] Priyadarshini M. Navarro G. Layden B. T. (2018). Gut microbiota: FFAR reaching effects on islets. Endocrinology 159, 2495–2505. doi: 10.1210/en.2018-00296, PMID: 29846565 PMC6692871

[ref112] Qi C. J. Zhang Q. Yu M. Xu J. P. Zheng J. Wang T.. (2016). Imbalance of fecal microbiota at newly diagnosed type 1 diabetes in Chinese children. Chin. Med. J. 129, 1298–1304. doi: 10.4103/0366-6999.182841, PMID: 27231166 PMC4894039

[ref113] Qin J. Li Y. Cai Z. Li S. Zhu J. Zhang F.. (2012). A metagenome-wide association study of gut microbiota in type 2 diabetes. Nature 490, 55–60. doi: 10.1038/nature11450, PMID: 23023125

[ref114] Qin J. Li R. Raes J. Arumugam M. Burgdorf K. S. Manichanh C.. (2010). A human gut microbial gene catalogue established by metagenomic sequencing. Nature 464, 59–65. doi: 10.1038/nature08821, PMID: 20203603 PMC3779803

[ref115] Que Y. Cao M. He J. Zhang Q. Chen Q. Yan C.. (2021). Gut bacterial characteristics of patients with type 2 diabetes mellitus and the application potential. Front. Immunol. 12:722206. doi: 10.3389/fimmu.2021.722206, PMID: 34484230 PMC8415158

[ref116] Rinott E. Youngster I. Yaskolka Meir A. Tsaban G. Zelicha H. Kaplan A.. (2021). Effects of diet-modulated autologous fecal microbiota transplantation on weight regain. Gastroenterology 160, 158–73.e10. doi: 10.1053/j.gastro.2020.08.041, PMID: 32860791 PMC7755729

[ref117] Roelofsen H. Priebe M. G. Vonk R. J. (2010). The interaction of short-chain fatty acids with adipose tissue: relevance for prevention of type 2 diabetes. Benefic. Microbes 1, 433–437. doi: 10.3920/BM2010.0028, PMID: 21831781

[ref118] Rosenbauer J. Herzig P. Giani G. (2008). Early infant feeding and risk of type 1 diabetes mellitus-a nationwide population-based case-control study in pre-school children. Diabetes Metab. Res. Rev. 24, 211–222. doi: 10.1002/dmrr.791, PMID: 17968982

[ref119] Ruiz-Canela M. Guasch-Ferré M. Toledo E. Clish C. B. Razquin C. Liang L.. (2018). Plasma branched chain/aromatic amino acids, enriched Mediterranean diet and risk of type 2 diabetes: case-cohort study within the PREDIMED trial. Diabetologia 61, 1560–1571. doi: 10.1007/s00125-018-4611-5, PMID: 29663011 PMC5988977

[ref120] Saeedi P. Petersohn I. Salpea P. Malanda B. Karuranga S. Unwin N.. (2019). Global and regional diabetes prevalence estimates for 2019 and projections for 2030 and 2045: results from the international diabetes federation diabetes atlas, 9(th) edition. Diabetes Res. Clin. Pract. 157:107843. doi: 10.1016/j.diabres.2019.107843, PMID: 31518657

[ref121] Säemann M. D. Böhmig G. A. Osterreicher C. H. Burtscher H. Parolini O. Diakos C.. (2000). Anti-inflammatory effects of sodium butyrate on human monocytes: potent inhibition of IL-12 and up-regulation of IL-10 production. FASEB J. 14, 2380–2382. doi: 10.1096/fj.00-0359fje, PMID: 11024006

[ref122] Saha S. Mara K. Pardi D. S. Khanna S. (2021). Long-term safety of fecal microbiota transplantation for recurrent Clostridioides difficile infection. Gastroenterology 160, 1961–9.e3. doi: 10.1053/j.gastro.2021.01.010, PMID: 33444573

[ref123] Sahuri-Arisoylu M. Brody L. P. Parkinson J. R. Parkes H. Navaratnam N. Miller A. D.. (2005). Reprogramming of hepatic fat accumulation and 'browning' of adipose tissue by the short-chain fatty acid acetate. Int. J. Obes. 40, 955–963. 10.1038/ijo.2016.23 26975441

[ref124] Savilahti E. Härkönen T. Savilahti E. M. Kukkonen K. Kuitunen M. Knip M. (2018). Probiotic intervention in infancy is not associated with development of beta cell autoimmunity and type 1 diabetes. Diabetologia 61, 2668–2670. doi: 10.1007/s00125-018-4738-4 30238182

[ref125] Schroeder B. O. Bäckhed F. (2016). Signals from the gut microbiota to distant organs in physiology and disease. Nat. Med. 22, 1079–1089. doi: 10.1038/nm.4185, PMID: 27711063

[ref126] Sedighi M. Razavi S. Navab-Moghadam F. Khamseh M. E. Alaei-Shahmiri F. Mehrtash A.. (2017). Comparison of gut microbiota in adult patients with type 2 diabetes and healthy individuals. Microb. Pathog. 111, 362–369. doi: 10.1016/j.micpath.2017.08.038, PMID: 28912092

[ref127] Shan Z. Sun T. Huang H. Chen S. Chen L. Luo C.. (2017). Association between microbiota-dependent metabolite trimethylamine-N-oxide and type 2 diabetes. Am. J. Clin. Nutr. 106, 888–894. doi: 10.3945/ajcn.117.157107, PMID: 28724646

[ref128] Shapiro H. Kolodziejczyk A. A. Halstuch D. Elinav E. (2018). Bile acids in glucose metabolism in health and disease. J. Exp. Med. 215, 383–396. doi: 10.1084/jem.20171965, PMID: 29339445 PMC5789421

[ref129] Shi H. Kokoeva M. V. Inouye K. Tzameli I. Yin H. Flier J. S. (2006). TLR4 links innate immunity and fatty acid-induced insulin resistance. J. Clin. Invest. 116, 3015–3025. doi: 10.1172/JCI28898, PMID: 17053832 PMC1616196

[ref130] Shreiner A. B. Kao J. Y. Young V. B. (2015). The gut microbiome in health and in disease. Curr. Opin. Gastroenterol. 31, 69–75. doi: 10.1097/MOG.0000000000000139, PMID: 25394236 PMC4290017

[ref131] Smits M. M. Fluitman K. S. Herrema H. Davids M. Kramer M. H. H. Groen A. K.. (2021). Liraglutide and sitagliptin have no effect on intestinal microbiota composition: a 12-week randomized placebo-controlled trial in adults with type 2 diabetes. Diabetes Metab. 47:101223. doi: 10.1016/j.diabet.2021.101223, PMID: 33429063

[ref132] Sommer F. Bäckhed F. (2013). The gut microbiota--masters of host development and physiology. Nat. Rev. Microbiol. 11, 227–238. doi: 10.1038/nrmicro2974, PMID: 23435359

[ref133] Sorini C. Cosorich I. Lo Conte M. De Giorgi L. Facciotti F. Lucianò R.. (2019). Loss of gut barrier integrity triggers activation of islet-reactive T cells and autoimmune diabetes. Proc. Natl. Acad. Sci. U. S. A. 116, 15140–15149. doi: 10.1073/pnas.1814558116, PMID: 31182588 PMC6660755

[ref134] Staudinger J. L. Goodwin B. Jones S. A. Hawkins-Brown D. MacKenzie K. I. LaTour A.. (2001). The nuclear receptor PXR is a lithocholic acid sensor that protects against liver toxicity. Proc. Natl. Acad. Sci. U. S. A. 98, 3369–3374. doi: 10.1073/pnas.051551698 11248085 PMC30660

[ref135] Stecher B. Hardt W. D. (2011). Mechanisms controlling pathogen colonization of the gut. Curr. Opin. Microbiol. 14, 82–91. doi: 10.1016/j.mib.2010.10.003, PMID: 21036098

[ref136] Stoddart L. A. Smith N. J. (2008). Milligan G. International Union of Pharmacology. LXXI. Free fatty acid receptors FFA1, −2, and −3: pharmacology and pathophysiological functions. Pharmacol. Rev. 60, 405–417. doi: 10.1124/pr.108.00802, PMID: 19047536

[ref137] Sturm A. Rilling K. Baumgart D. C. Gargas K. Abou-Ghazalé T. Raupach B.. (2005). Escherichia coli Nissle 1917 distinctively modulates T-cell cycling and expansion via toll-like receptor 2 signaling. Infect. Immun. 73, 1452–1465. doi: 10.1128/IAI.73.3.1452-1465.2005, PMID: 15731043 PMC1064918

[ref138] Su L. Hong Z. Zhou T. Jian Y. Xu M. Zhang X.. (2022). Health improvements of type 2 diabetic patients through diet and diet plus fecal microbiota transplantation. Sci. Rep. 12:1152. doi: 10.1038/s41598-022-05127-9, PMID: 35064189 PMC8782834

[ref139] Sudo N. Yu X. N. Aiba Y. Oyama N. Sonoda J. Koga Y.. (2002). An oral introduction of intestinal bacteria prevents the development of a long-term Th2-skewed immunological memory induced by neonatal antibiotic treatment in mice. Clin. Exp. Allergy 32, 1112–1116. doi: 10.1046/j.1365-2222.2002.01430.x, PMID: 12100062

[ref140] Sumida K. Han Z. Chiu C. Y. Mims T. S. Bajwa A. Demmer R. T.. (2022). Circulating microbiota in Cardiometabolic disease. Front. Cell. Infect. Microbiol. 12:892232. doi: 10.3389/fcimb.2022.892232, PMID: 35592652 PMC9110890

[ref141] Sun J. Furio L. Mecheri R. van der Does A. M. Lundeberg E. Saveanu L.. (2015). Pancreatic β-cells limit autoimmune diabetes via an immunoregulatory antimicrobial peptide expressed under the influence of the gut microbiota. Immunity 43, 304–317. doi: 10.1016/j.immuni.2015.07.013, PMID: 26253786

[ref142] Sun L. Xie C. Wang G. Wu Y. Wu Q. Wang X.. (2018). Gut microbiota and intestinal FXR mediate the clinical benefits of metformin. Nat. Med. 24, 1919–1929. doi: 10.1038/s41591-018-0222-4, PMID: 30397356 PMC6479226

[ref143] Tai N. Peng J. Liu F. Gulden E. Hu Y. Zhang X.. (2016). Microbial antigen mimics activate diabetogenic CD8 T cells in NOD mice. J. Exp. Med. 213, 2129–2146. doi: 10.1084/jem.20160526, PMID: 27621416 PMC5030808

[ref144] Takewaki F. Nakajima H. Takewaki D. Hashimoto Y. Majima S. Okada H.. (2021). Habitual dietary intake affects the altered pattern of gut microbiome by Acarbose in patients with type 2 diabetes. Nutrients 13. doi: 10.3390/nu13062107, PMID: 34205413 PMC8235473

[ref145] Thaiss C. A. Levy M. Grosheva I. Zheng D. Soffer E. Blacher E.. (2018). Hyperglycemia drives intestinal barrier dysfunction and risk for enteric infection. Science 359, 1376–1383. doi: 10.1126/science.aar3318, PMID: 29519916

[ref146] The Integrative Human Microbiome Project (2019). The Integrative Human Microbiome Project. Nature 569, 641–648. doi: 10.1038/s41586-019-1238-8 31142853 PMC6784865

[ref147] Thomas C. Gioiello A. Noriega L. Strehle A. Oury J. Rizzo G.. (2009). TGR5-mediated bile acid sensing controls glucose homeostasis. Cell Metab. 10, 167–177. doi: 10.1016/j.cmet.2009.08.001, PMID: 19723493 PMC2739652

[ref148] Tian Y. Nichols R. G. Cai J. Patterson A. D. Cantorna M. T. (2018). Vitamin a deficiency in mice alters host and gut microbial metabolism leading to altered energy homeostasis. J. Nutr. Biochem. 54, 28–34. doi: 10.1016/j.jnutbio.2017.10.011, PMID: 29227833 PMC5866754

[ref149] Tlaskalová-Hogenová H. Stěpánková R. Kozáková H. Hudcovic T. Vannucci L. Tučková L.. (2011). The role of gut microbiota (commensal bacteria) and the mucosal barrier in the pathogenesis of inflammatory and autoimmune diseases and cancer: contribution of germ-free and gnotobiotic animal models of human diseases. Cell. Mol. Immunol. 8, 110–120. doi: 10.1038/cmi.2010.67, PMID: 21278760 PMC4003137

[ref150] Tolhurst G. Heffron H. Lam Y. S. Parker H. E. Habib A. M. Diakogiannaki E.. (2012). Short-chain fatty acids stimulate glucagon-like peptide-1 secretion via the G-protein-coupled receptor FFAR2. Diabetes 61, 364–371. doi: 10.2337/db11-1019, PMID: 22190648 PMC3266401

[ref151] Uusitalo U. Liu X. Yang J. Aronsson C. A. Hummel S. Butterworth M.. (2016). Association of Early Exposure of probiotics and islet autoimmunity in the TEDDY study. JAMA Pediatr. 170, 20–28. doi: 10.1001/jamapediatrics.2015.2757, PMID: 26552054 PMC4803028

[ref152] Vallianou N. G. Stratigou T. Tsagarakis S. (2018). Microbiome and diabetes: where are we now? Diabetes Res. Clin. Pract. 146, 111–118. doi: 10.1016/j.diabres.2018.10.008, PMID: 30342053

[ref153] van Nood E. Vrieze A. Nieuwdorp M. Fuentes S. Zoetendal E. G. de Vos W. M.. (2013). Duodenal infusion of donor feces for recurrent Clostridium difficile. N. Engl. J. Med. 368, 407–415. doi: 10.1056/NEJMoa1205037, PMID: 23323867

[ref154] Velloso L. A. Folli F. Saad M. J. (2015). TLR4 at the crossroads of nutrients, gut microbiota, and metabolic inflammation. Endocr. Rev. 36, 245–271. doi: 10.1210/er.2014-1100, PMID: 25811237

[ref155] Vindigni S. M. Surawicz C. M. (2017). Fecal microbiota transplantation. Gastroenterol. Clin. N. Am. 46, 171–185. doi: 10.1016/j.gtc.2016.09.012 28164849

[ref156] Vrancken G. Gregory A. C. Huys G. R. B. Faust K. Raes J. (2019). Synthetic ecology of the human gut microbiota. Nat. Rev. Microbiol. 17, 754–763. doi: 10.1038/s41579-019-0264-8, PMID: 31578461

[ref157] Vrieze A. Van Nood E. Holleman F. Salojärvi J. Kootte R. S. Bartelsman J. F.. (2012). Transfer of intestinal microbiota from lean donors increases insulin sensitivity in individuals with metabolic syndrome. Gastroenterology 143, 913–6.e7. doi: 10.1053/j.gastro.2012.06.031, PMID: 22728514

[ref158] Walker W. A. (2017). The importance of appropriate initial bacterial colonization of the intestine in newborn, child, and adult health. Pediatr. Res. 82, 387–395. doi: 10.1038/pr.2017.111, PMID: 28426649 PMC5570628

[ref159] Wang H. Lu Y. Yan Y. Tian S. Zheng D. Leng D.. (2019). Promising treatment for type 2 diabetes: fecal microbiota transplantation reverses insulin resistance and impaired islets. Front. Cell. Infect. Microbiol. 9:455. 32010641 10.3389/fcimb.2019.00455 PMC6979041

[ref160] Wang H. B. Wang P. Y. Wang X. Wan Y. L. Liu Y. C. (2012). Butyrate enhances intestinal epithelial barrier function via up-regulation of tight junction protein Claudin-1 transcription. Dig. Dis. Sci. 57, 3126–3135. doi: 10.1007/s10620-012-2259-4, PMID: 22684624

[ref161] Watanabe M. Houten S. M. Mataki C. Christoffolete M. A. Kim B. W. Sato H.. (2006). Bile acids induce energy expenditure by promoting intracellular thyroid hormone activation. Nature 439, 484–489. doi: 10.1038/nature04330, PMID: 16400329

[ref162] Wu H. Esteve E. Tremaroli V. Khan M. T. Caesar R. Mannerås-Holm L.. (2017). Metformin alters the gut microbiome of individuals with treatment-naive type 2 diabetes, contributing to the therapeutic effects of the drug. Nat. Med. 23, 850–858. doi: 10.1038/nm.4345, PMID: 28530702

[ref163] Xia T. Lai W. Han M. Han M. Ma X. Zhang L. (2017). Dietary ZnO nanoparticles alters intestinal microbiota and inflammation response in weaned piglets. Oncotarget 8, 64878–64891. doi: 10.18632/oncotarget.17612, PMID: 29029398 PMC5630298

[ref164] Xiong Y. Miyamoto N. Shibata K. Valasek M. A. Motoike T. Kedzierski R. M.. (2004). Short-chain fatty acids stimulate leptin production in adipocytes through the G protein-coupled receptor GPR41. Proc. Natl. Acad. Sci. U. S. A. 101, 1045–1050. doi: 10.1073/pnas.2637002100, PMID: 14722361 PMC327148

[ref165] Xu Q. Ni J. J. Han B. X. Yan S. S. Wei X. T. Feng G. J.. (2021). Causal relationship between gut microbiota and autoimmune diseases: a two-sample Mendelian randomization study. Front. Immunol. 12:746998. doi: 10.3389/fimmu.2021.746998 35140703 PMC8819003

[ref166] Yatsunenko T. Rey F. E. Manary M. J. Trehan I. Dominguez-Bello M. G. Contreras M.. (2012). Human gut microbiome viewed across age and geography. Nature 486, 222–227. doi: 10.1038/nature11053, PMID: 22699611 PMC3376388

[ref167] Zhang Y. Lee F. Y. Barrera G. Lee H. Vales C. Gonzalez F. J.. (2006). Activation of the nuclear receptor FXR improves hyperglycemia and hyperlipidemia in diabetic mice. Proc. Natl. Acad. Sci. U. S. A. 103, 1006–1011. doi: 10.1073/pnas.0506982103, PMID: 16410358 PMC1347977

[ref168] Zhao L. Lou H. Peng Y. Chen S. Zhang Y. Li X. (2019). Comprehensive relationships between gut microbiome and faecal metabolome in individuals with type 2 diabetes and its complications. Endocrine 66, 526–537. doi: 10.1007/s12020-019-02103-8, PMID: 31591683

[ref169] Zhao L. Zhang F. Ding X. Wu G. Lam Y. Y. Wang X.. (2018). Gut bacteria selectively promoted by dietary fibers alleviate type 2 diabetes. Science 359, 1151–1156. doi: 10.1126/science.aao5774, PMID: 29590046

[ref170] Zhou D. Chen Y. W. Zhao Z. H. Yang R. X. Xin F. Z. Liu X. L.. (2018). Sodium butyrate reduces high-fat diet-induced non-alcoholic steatohepatitis through upregulation of hepatic GLP-1R expression. Exp. Mol. Med. 50, 1–12. doi: 10.1038/s12276-018-0183-1, PMID: 30510243 PMC6277380

[ref171] Zhou D. Pan Q. Xin F. Z. Zhang R. N. He C. X. Chen G. Y.. (2017). Sodium butyrate attenuates high-fat diet-induced steatohepatitis in mice by improving gut microbiota and gastrointestinal barrier. World J. Gastroenterol. 23, 60–75. doi: 10.3748/wjg.v23.i1.60, PMID: 28104981 PMC5221287

[ref172] Zhou Z. Sun B. Yu D. Zhu C. (2022). Gut microbiota: an important player in type 2 diabetes mellitus. Front. Cell. Infect. Microbiol. 12:834485. doi: 10.3389/fcimb.2022.834485, PMID: 35242721 PMC8886906

[ref173] Zhou H. Sun L. Zhang S. Zhao X. Gang X. Wang G. (2021). The crucial role of early-life gut microbiota in the development of type 1 diabetes. Acta Diabetol. 58, 249–265. doi: 10.1007/s00592-020-01563-z, PMID: 32712802

[ref174] Zhu T. Goodarzi M. O. (2020). Metabolites linking the gut microbiome with risk for type 2 diabetes. Curr. Nutr. Rep. 9, 83–93. doi: 10.1007/s13668-020-00307-3, PMID: 32157661 PMC7282969

